# Every layer of quadriceps tendon’s central and medial portion offers similar tensile properties than Hamstrings or Ilio-Tibial Band Grafts

**DOI:** 10.1186/s40634-020-00261-7

**Published:** 2020-07-10

**Authors:** Matthieu Chivot, Charles Pioger, Jérémy Cognault, Akash Sharma, Regis Pailhé, Etienne Cavaignac, Matthieu Ollivier, Christophe Jacquet

**Affiliations:** 1 Aix-Marseille University, CNRS, ISM UMR 7287, 13288, Cedex 09 Marseille, France; 2 Clinique du Parc, 69006 Lyon, France; 3 Department of Orthopaedic Surgery and Sport Traumatology, Grenoble South Teaching Hospital, 38130 Echirolles, France; 4 Department of Orthopedic Surgery and Trauma, Hôpital Pierre Paul Riquet, Toulouse, France; 5 Department of Orthopedic surgery and Traumatology, University institute of movement and locomotion, St. Marguerite Hospital, 270 Boulevard Sainte Marguerite, 29 13274 Marseille, BP France

**Keywords:** Quadriceps tendon, Mechanical properties, Ligament reconstruction, Hamstring, Patellar tendon

## Abstract

**Purpose:**

The aim of our cadaveric study was to compare the mechanical properties of different parts of the quadriceps’ tendon in a load to failure analysis as compared to three other, and most common types of grafts that are used to perform ligament’s reconstruction.

**Methods:**

Ten fresh-frozen cadavers (5 women, 5 men) were selected from our anatomical department. Mean age at death was 64 years (48–87 years). Tendons were harvested to prepare (1) different quadriceps tendon’s specimens: lateral portion (QTlat), medial portion superficial layer (QTMsup) and deep layer (QTMdeep) and central portion superficial (QTCsup) and deep layers (QTCdeep) (2) Patellar Tendon (PT), (3) Gracilis+Semi-Tendinosus specimens (GST). Specimens were stored at − 40 °C in a freezing solution. Specimens were securely attached to a dedicated loading platform, measurements were done using a validated software. Load to failure testing was then carried out. Young’s Elastic moduli, ultimate Stress (MPa) and Deformation (%) were analysed.

**Results:**

The elastic moduli of the PT was significantly higher than all other grafts, all medial and central QT layers (superficial and deep) were significantly higher than its lateral part (QTlat). In terms of Ultimate Stress, all grafts were significantly greater than QTlat, PT and GST were significantly superior to QT central portions and to ITB but there did not differ with the medial portion of QT. ITB ultimate stress values were significantly higher than QTlat. The ultimate deformations of all grafts were similar.

**Conclusions:**

This study provides reference values in in order to characterize different parts of the QT that presents anatomically and Mechanically with complex characteristics. Every Layer of Quadriceps Tendon’s Central and Medial Portion Offered Similar Mechanical Properties than Two Strand Hamstrings or Ilio-Tibial Band.

## Introduction

The majority of the tendon autografts demonstrate themselves to be safe and clinically efficient for knee ligament reconstruction^1^. Their use is often based on a combination of factors including; their structural properties, size, donor site morbidity, graft availability, patients’ activity level, perceived functional outcome, and ultimately, the surgeons preference [7–35]. Understanding the mechanical properties and thus the intrinsic behavior is a necessary adjunct to help guiding graft choice.

These properties are independent of the size, volume or the influence of their attachment sites Whilst, the mechanical properties of the patellar tendon are well established within the literature [2–4, 12, 14, 25, 30, 33], there are relatively fewer studies on the mechanical properties of the hamstrings [1, 4, 12, 25], ITB [4, 11, 25, 28], and even less so on the quadriceps tendon [22, 25, 30, 34].

More, divided use of quadriceps tendon layers have been advocated to mimic ACL’s anatomy the graft being either split in the sagittal [18] or coronal planes [23]. Properties of an intact quadriceps grafts have been already quantified [22, 30, 33, 34] and recently the use of coronal and sagittal plane splitting for a double-bundle ACL reconstruction has been shown to result in similar tensile properties between the graft halves regardless of the splitting plane [23].

However, data are missing regarding tensile properties of quadriceps tendon dissected in multiple layers and parts.

Therefore, the aim of this cadaveric study was to compare the mechanical properties of different portions of the quadriceps tendon in a load to failure analysis as compared to three other types of grafts that can be used to perform ligament reconstruction. Our hypothesis was that the mechanical properties of different layers of the quadriceps tendon were similar to those of graft commonly used for ligaments reconstructions.

## Methods

### Specimen preparation

Ten fresh-frozen cadavers (5 women, 5 men) were selected from our anatomical department. Mean age at death was 64 years (range: 48 to 87 years). The cadavers were stored at − 8 °C. A single knee from each specimen was used to prepare specimens, and the 10 cadaveric knees were deeply evaluated for signs of bony or articular disease and/or surgery. Selection was based on age, absence of surgical history and absence of knee osteoarthritis (X-ray evaluation). Any knee specimens meeting one of the following exclusion criteria were not used: wounds or macroscopic signs of intra-articular lesions wounds or old lesions of the quadriceps and/or other tendons, evidence of patella fracture.

Institutional review board was not consulted as specimens came from donations to the anatomy laboratory and pathology department. Anatomical and biomechanical labs scientific committees validated our study protocol and ensured ethical use of de-identified specimens (N° 2019–015724-11)

**Fig. 1:**
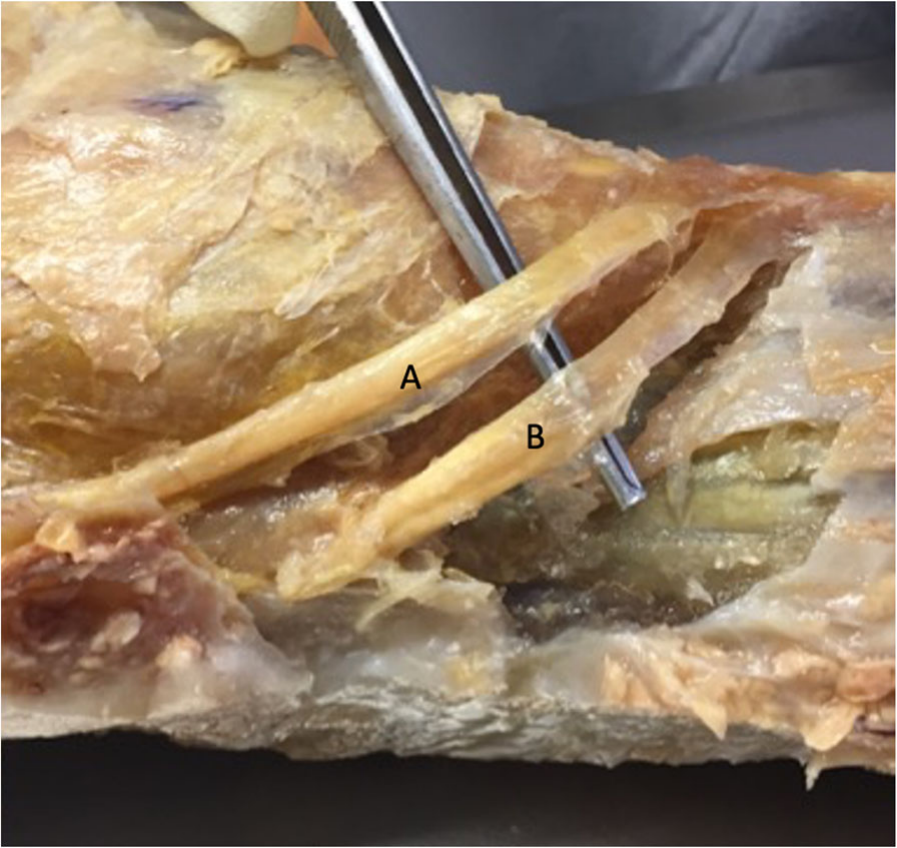
Hamstring specimens Preparation/ A = Gracilis tendon / B = Semi-Tendinosus tendon

.All grafts were harvested at our university’s anatomy laboratory. All skin tissues were excised to allow identification of different structures. The semitendinosus was identified in the lower and medial part of anterior tibial tuberosity after the sartorius fascia was opened (Fig. 1). These tendons were harvested from their muscle bodies with an open tendon stripper, and then dissected from their tibial attachment at the periosteum. All core knee muscles and structures were not dissected or disturbed from their anatomic position, including the patella, patellar tendon, and the quadriceps tendon. The knee was then dissected to harvest the Iliotibial band. This was carried out using protocols described by Christel and Djian [6]. Once the ITB was identified it was separated from the biceps femoris tendon, and then a graft of 10 cm long by 10 mm wide was harvested by detaching it from Gerdy’ s tubercle.

For Patellar tendon harvesting, only the central potion was harvested to obtain a 1 cm large tendinous graft without any bone plug.

For quadriceps tendon harvesting, the entire tendon was exposed from the superior pole of the patella to the muscular bellies of the vastus medialis (VM), the vastus lateralis (VL), and rectus femoris (RF).

**Fig. 2:**
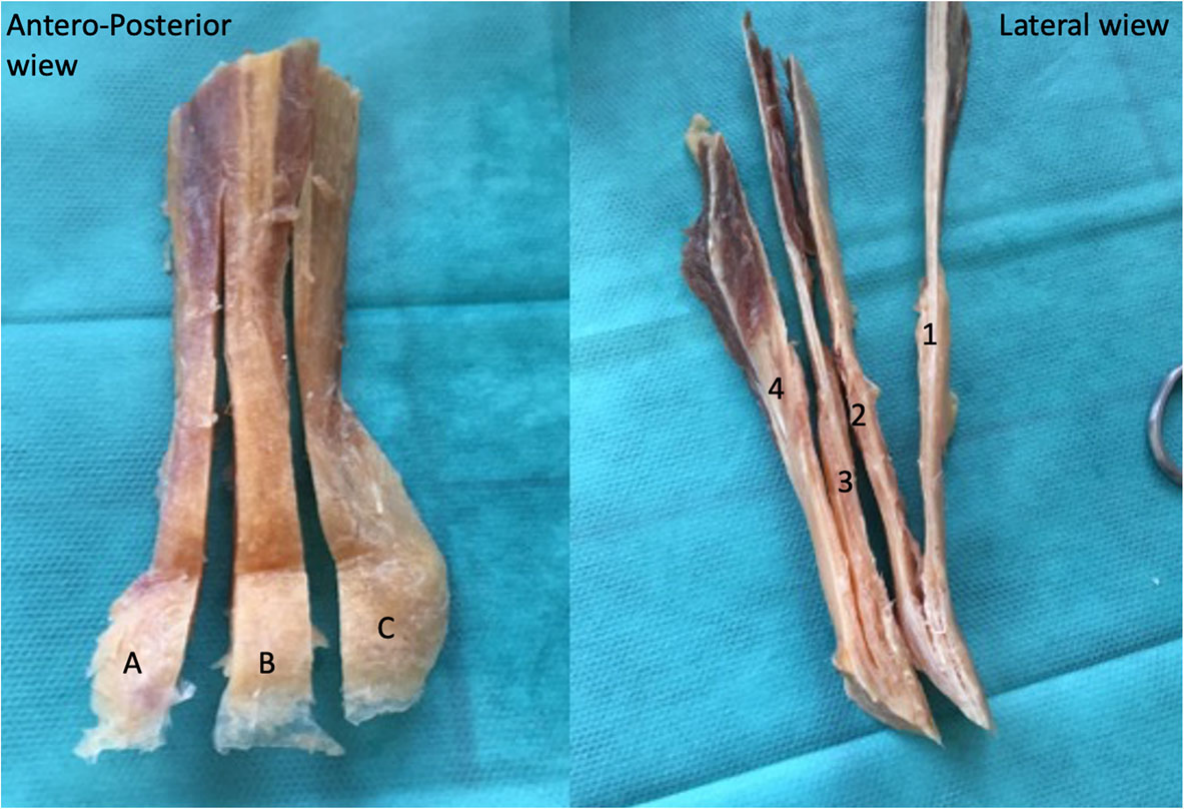
Quadriceps Tendon specimens Preparation / A = QT Lateral /B = QT Central / C = QT Medial/ 1 = Superficial layer of the medial zone / 2 = Deep layer of the medial zone / 3 = Superficial layer of the central zone / 5 = Deep layer of the central zone

Harvesting was performed by using a No. 11 scalpel blade incising along the axis of the tendon fibers at the junction with VM and VL. Both incisions were extended distally to the periosteum on the patella. Proximal portion was detached at its junction with the rectus femoris at mean 13.8 cm proximally from the patella (9.5 to 18.2 cm). Only the two most superficial layers were harvested to avoid entering the joint. The dissection was then extended from the patella proximally [10]. The quadriceps tendon once harvested was measured to prepare 5 different quadriceps tendon’s specimens (Fig. 2), and a further 3 strips of equal widths were separated according to their position (lateral, central, medial). Defining 3 zones was justified by the complex architecture of the quadriceps tendon which is organized in 3 layers with the fascial extensions of the muscles of the quadriceps which meet near the patella to form an “Onion-like” structure [27]. Only the 2 most superficial layers interested us, they are separated by a thin layer of fat tissue, they merge as previously demonstrated by Grob [10] in 2 points at a fairly constant distance from the proximal pole of the patella.

These layers were then separated using a No. 11 knife and dissected to harvest two distinct layers (superficial and deep) which were then measured with an electronic calibrator (Mitutoyo®) with an accuracy of 0.01 mm. All measurements were performed by the same person five times, then cross-sectional was calculated the mean was taken.

Therefore, from the quadriceps tendon harvest, we were able to produce 5 samples, a deep and superficial medial (QTMsup and QTMdeep), a superficial and deep central (QTCsup and QTCdeep) and total lateral (QTlat).

The gracilis+semi-tendinosus (GST) specimen was folded into two and each end was sutured to itself using a No. 2 Vicryl (polyglactin 910), to form a two-strand graft [32].

The ITB did not require any special preparation.

Specimens were stored at − 40 °C in a freezing solution containing saline and 10% dimethyl sulfoxide. It has been shown that this storage protocol does not alter the biomechanical properties of tendons [24]. They were removed from the freezer and thawed at room temperature (21 °C) for at least 12 h before experimentation.

### Testing protocol

All isolated grafts were tested without any bone attachment. Ends of the grafts were placed in two self-gripping traction machines.

**Fig. 3:**
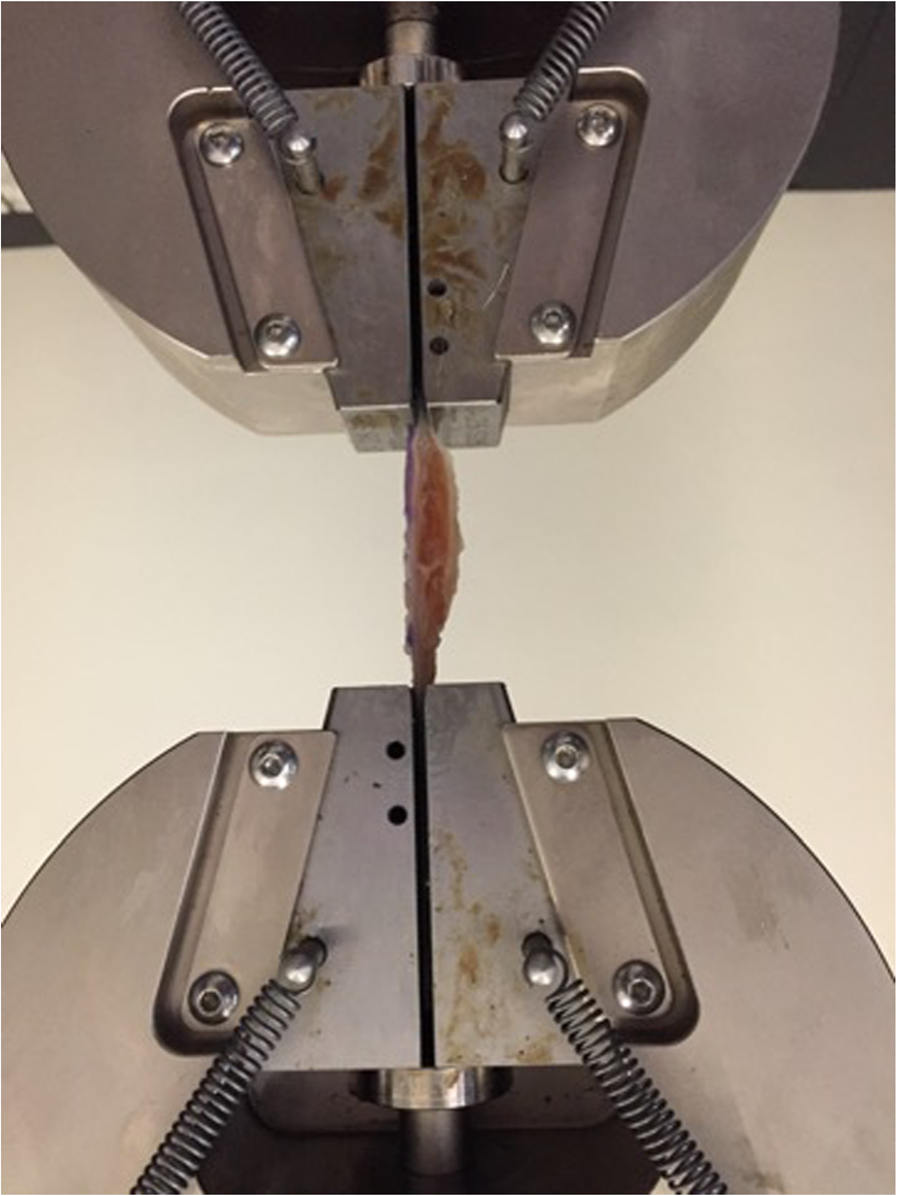
Load to failure testing of a Patellar tendon specimen

Each jaws-tendon-jaws assembly was placed in a traction / compression device (Instrom 5566, Instron, Canton, MA) to apply axial tensile loads (Fig. 3) [16]. Measurements were made using the system software (BlueHill, Instrom SA France, Elancourt, France).

To minimize the bias in the preparation of the specimen, a digital caliper (Absolute.

Digimatic®, MitutoyoTM, Kanagawa,Japan) with a precision of U = 0 .001 mm was used in.

order to measure each samples thickness, length and width under a 10 N load [16]. These measurements allow section’s size calculation for each sample thus permitting stresses and strains estimation.

Each sample was preloaded at 10 N for 30 s. The interface of each specimen/jaw was marked with China ink to monitor potential sliding. All specimens were subjected to a tensile test with a progression of 10 mm/min to failure. The measurements used followed a validated standard test protocol [26, 37]. Maximum failure load (N) and elongation at failure (mm) were automatically measured by the software during the test. The linear stiffness (N / mm), the stress (N / mm2), the deformation and the Young’s modulus were calculated as set out below. The preparation, preservation, graft fixation and measurements were performed by the same individuals.

The main characteristics of the samples are summarized in Table 1.

**Table 1:** Physical characteristic of the specimens

	Length between jaws (mm)	Mean diameter (mm)	Mean thickness (mm)
**GST**	43,42 (39,12-42,18)	7,34 (4,14-9,60)	_
**PT**	37,72 (33,13-42,27)	_	8,43 (4,19-12,3)
**ITB**	39,03 (32,20-42,42)	_	2,63 (1,80-3,13)
**QTLat**	23,46 (26,28-38,31)	_	3,73 (2,57-4,40)
**QTCsup**	39,13 (24,53-41,29)	_	3,65 (1,95-4,89)
**QTCdeep**	37,53 (28,52-42,78)	_	2,92 (1,49-3,39)
**QTMdeep**	41,71 (23,65-40,33)	_	3,63 (1,56-4,49)
**QTMsup**	40,81 (25,83-39,62)	_	3,82 (2,62-5,60)

*QTlat* lateral, *QTMsup* Superficial layer of the medial zone, *QTMdeep* Deep layer of the of the median zone, *QTCsup* Superficial layer of the central zone, *QTCdeep* Deep layer of the central zone, *PT* Patellar Tendon, *GST* Gracilis+Semi-Tendinosus, *ITB* Iliotibial band

### Statistical analysis

Calculations were done using Excel 2016 software (Microsoft, Redmond, WA). Statistical analyses were performed using PASW Statistics version 20 (SPSS, IBM Inc., Chicago, Illinois). The normal distribution of measured variables was verified using the Kolmogorov-Smirnov test and the Levene test to ensure that the conditions were met for parametric tests. A post-hoc analysis estimated that our sample size (7 specimens by group) allowed us to appreciate ultimate stress and elastic Modulus specimens’ differences superior to 10+/− 10% with a sufficient statistical power.

The significance level was set at *p* < 0.05. The descriptive analysis consisted of mean, median and standard deviation values. After testing differences in terms of mechanical parameters using ANOVA testing a deeper comparative analysis was performed using a pairwise comparison to distinguish differences among each type of grafts.

## Results

80 specimens were harvested and created; 4 samples were excluded from the results because they slipped out of the jaws during loading analysis (Table 2).

**Table 2:** Mechanical Testing results (Mean values and standard deviation) in term of Elastic moduli, Ultimate stress and Deformation

	Elastic Modulus (Mpa)	Ultimate Stress (Mpa)	Ultimate Deformation (%)
GST	89 ± 101,3	99,3 ± 74,4	36 ± 5,3
PT	176 ± 119	140,9 ± 9,3	12 ± 4,6
QTMdeep	100 ± 65	95,2 ± 81	19 ± 9,8
QTMsup	86,5 ± 69	79,8 ± 50	14 ± 5,8
QTCdeep	79,6 ± 51	79,1 ± 74	21 ± 5,6
QTCsup	56,5 ± 46	75,1 ± 55	19 ± 9,9
ITB	48,8 ± 37	23 ± 10	24 ± 15
QTLat	19,9 ± 11	42,6 ± 4	24 ± 23

Specimens: *QTlat* lateral, *QTMsup* Superficial layer of the medial zone, *QTMdeep* Deep layer of the of the median zone, *QTCsup* Superficial layer of the central zone, *QTCdeep* Deep layer of the central zone, *PT* Patellar Tendon, *GST* Gracilis+Semi-Tendinosus *ITB* Iliotibial band

The elastic modulus of the PT was significantly higher than all other grafts (176 ± 119 MPa). All QT medial and central layers (superficial and deep) were significantly higher than its lateral part QTlat. However, they were not significantly superior to ITB. (Table 3).

**Table 3:** Comparison between specimens in terms of Elastic Moduli (Mean values)

Specimens	GST	PT	QTMdeep	QTMsup	QTCdeep	QTCsup	QTlat	ITB
**GST**	─	Δ87 ***p <*** 0.0001*	Δ 11 ***p*** **=** 0.01*	Δ 2.5 *p* = 0.09	Δ 9.4 *p* = 0.07	Δ32.5 *p* = 0.001*	Δ69.1 ***p*** = 0.0007*	Δ40.2 ***p*** = 0.0009*
**PT**		─	Δ76 ***p <*** 0.0001 *	Δ89.5 ***p <*** 0.0001 *	Δ96.4 ***p <*** 0.0001*	Δ119.5 ***p <*** 0.0001*	Δ156.1 ***p <*** 0.0001*	Δ127.2 ***p <*** 0.0001*
**QTM deep**			─	Δ13.5 p = 0.01*	Δ20.4 ***p*** = 0.004*	Δ43.5 ***p*** = 0.0009*	Δ80.1 ***p*** = 0.0006*	Δ51.2 ***p =*** 0.0007*
**QTM sup**				─	Δ6.9 *p =* 0.09	Δ30 ***p*** = 0.001*	Δ66.6 ***p =*** 0.0006*	Δ37.7 ***p*** = 0.001*
**QTC deep**					─	Δ23.1 ***p =*** 0.04*	Δ59.7 ***p*** = 0.0004*	Δ30.8 ***p*** = 0.002*
**QTCsup**						─	Δ36.6 ***p =*** 0.01*	Δ 7.7 *p* = 0.06
**QTlat**							─	Δ28.9 ***p =*** 0.002*

Specimens:*QTlat* lateral, *QTMsup* Superficial layer of the medial zone, *QTMdeep* Deep layer of the of the median zone, *QTCsup* Superficial layer of the central zone, *QTCdeep* Deep layer of the central zone, *PT* Patellar Tendon, *GST* Gracilis+Semi-Tendinosus, *ITB* Iliotibial band

*a pairwise comparison exhibited significant differences

Regarding the ultimate stress, all grafts exhibit superior values than QTlat, PT and GST were significantly superior to QT’s central portion (both superficial and deep layers) and to ITB but there was no significant difference with the deep and superficial layers of QT’s medial portion. ITB ultimate stress was significantly higher than QTlat (Table 4).

**Table 4:** Comparison between specimens in terms of Ultimate Stress (Mean values)

Specimen	GST	PT	QTMdeep	QTMsup	QTCdeep	QTCsup	QTlat	ITB
**GST**	─	Δ41.6 ***p =*** 0.01*	Δ4.1 *p* = 0.4	Δ19.5 ***p*** = 0.04 *	Δ20.2 ***p =*** 0.04*	Δ24.2 ***p*** = 0.03*	Δ56.7 ***p*** = 0.007*	Δ76.3 ***p =*** 0.001*
**PT**		─	Δ45.7 ***p =*** 0.01*	Δ61.1 ***p*** = 0.006 *	Δ61.8 ***p =*** 0.006*	Δ65.8 ***p*** = 0.005*	Δ98.3 ***p <*** 0001*	Δ117.9 ***p <*** 0001*
**QTM deep**			─	Δ15.4 *p =* 0.06	Δ16.1 *p =* 0.06	Δ20.1 ***p =*** 0.04*	Δ 52.6 ***p*** = 0.008*	Δ72.2 ***p*** = 0.003*
**QTM sup**				─	Δ0.7 *p =* 0.4	Δ 4.7 *p =* 0.2	Δ37.2 ***p =*** 0.01*	Δ56.8 ***p =*** 0.006*
**QTC deep**					─	Δ 4.0 *p* = 0.8	Δ 36.5 ***p*** = 0.02*	Δ56.1 ***p =*** 0.007*
**QTCsup**						─	Δ32.5 ***p =*** 0.03*	Δ52.1 ***p =*** 0.008*
**QTlat**							─	Δ19.6 ***p =*** 0.04*

Specimens:*QTlat* lateral, *QTMsup,* Superficial layer of the medial zone, *QTMdeep* Deep layer of the of the median zone, *QTCsup* Superficial layer of the central zone, *QTCdeep* Deep layer of the central zone, *PT* Patellar Tendon, *GST* Gracilis+Semi-Tendinosus, *ITB* Iliotibial band

* a pairwise comparison exhibited significant differences

Significant differences were found regarding mean deformation between PT vs QTCSup and PT vs QTlat respectively, as well as between QTMDeep vs QTCSup (Table 5).

**Table 5:** Comparison between specimens in terms of Ultimate Deformation (Mean values)

Specimen	GST	PT	QTMdeep	QTMsup	QTCdeep	QTCsup	QTlat	ITB
**GST**	─	Δ24 ***p <*** 0..0001*	Δ17 ***p*** < 0..0001*	Δ22 ***p <*** 0..0001*	Δ15 ***p =*** 0.0009*	Δ17 ***p <*** 0..0001*	Δ12 ***p =*** 0..001*	Δ12 ***p =*** 0.001*
**PT**		─	Δ7 ***p =*** 0.03*	Δ2 *p =* 0.08	Δ9 ***p =*** 0.04 *	Δ7 ***p =*** 0.03*	Δ12 ***p =*** 0.001*	Δ12 ***p =*** 0.001*
**QTM deep**			─	Δ5 *p =* 0.05	Δ2 *p* = 0.1	Δ0 p: N/A	Δ 5 *p* = 0.05	Δ5 *p =* 0.06
**QTM sup**				─	Δ7 ***p =*** 0.04*	Δ 5 ***p =*** 0.04*	Δ10 ***p =*** 0.005 *	Δ10 ***p =*** 0.008*
**QTC deep**					─	Δ2 *p =* 0.8	Δ 3 *p =* 0.4	Δ3 *p =* 0.4
**QTCsup**						─	Δ5 *p =* 0.06	Δ5 *p =* 0.05
**QTlat**							─	Δ0 p: N/A

Specimens:*QTlat* lateral, *QTMsup* Superficial layer of the medial zone, *QTMdeep* Deep layer of the of the median zone, *QTCsup* Superficial layer of the central zone, *QTCdeep* Deep layer of the central zone, *PT* Patellar Tendon, *GST* Gracilis+Semi-Tendinosus, *ITB* Iliotibial band

* a pairwise comparison exhibited significant differences

## Discussion

The main finding of this study was that mechanical properties of the QT superficial and deep layers in its central and medial portion are equal and/or superior to those of Hamstrings and ITB but remain lower in terms of mechanical resistance to those patellar tendons. These results assume that differing quadriceps tendon layers possess mechanical properties compatible with an anterior cruciate ligament reconstruction.

QT is a graft already used and validated for reconstructions of cruciate ligaments [17]. It can therefore be used as a transplant when its central part is taken almost completely. Harris et al. [13] measured division between the two tendons at 6 cm proximal to the insertion of the patella, which corresponded to our observations. The central and medial parts were thicker than the outer part. Lippe et al. [21] confirmed in their anatomical study the observations of Harris et al. [13] who described a surface asymmetry of QT with a lower insertion of VM compared to VL. Potage et al. [27] confirmed that if the surgeon wants a thicker graft, he should harvest only central and medial bands of the QT, which corresponded to our sample thicknesses.

Staubli et al. [33] compared properties of QT with those of patellar tendon, the samples corresponded to a band 10 mm wide with a total thickness of the medial quadriceps tendon and its retained bone insertion. They found an ultimate stress of overall QT sample to be 33.6 +/− 8.1 N/mm2 versus 53.4 +/− 7.2 N/mm2 for PT. Miller et al. [23] analysed in their study split QT specimen in two parts, 10 tendons separated in sagittal plane and coronal plane, the author did not find any significant differences in biomechanical parameters between the two halves or in the cleavage plane. They found a maximum load of 445 +/− 210 N for the different parts. It seems that the complex organization of the QT when it is fully preserved gives it an exponential resistance.

Herbort et al. [15] used 10 mm × 3 mm thick QT samples to reconstruct the medial patellofemoral ligament (MPFL). They found a maximum load before failure at 205 N +/− 77.8 N. In our study, the QTCsup sample corresponded to the same sample as that of Herbort et al. [15]: we found an ultimate stress before failure of 75,1 ± 55 N/mm2. But our results are expressed in N/mm2 (stress and not load before failure) to account for the difference in cross-sectional area of the samples. Difference between our results, even if it is significant, can be explained by our test protocols being slightly different. Samples were cycled 1000 times between 5 and 50 N whilst in our study our specimens were preloaded at 10 N for 30 s.

Finally, The results of the different studies on material properties vary markedly, thus making comparisons difficult. Donor age, strain rate, biologic variability, cross-sectional area calculation, and testing protocol are variables that can explain the differences between studies.

Some limitations can be attributed to this study. Firstly, the tensile test was performed on frozen/thawed grafts, but it is important to note that it has been shown that the mechanical properties of tendons are not affected by freezing if less than 3 cycles of freezing–thawing are performed [4, 38]. Secondly, the fixation can influence the results of these tensile tests. Jaws made from resin or cryoclamps are difficult to use and their use has not been validated [5, 29]. Shi and al [31]. validated the use of serrated jaws for ligament autograft fixation after comparing them to other type of jaws. It is this type of jaw that that has been used in this study.

Thirdly, the tensile tests were performed with dissected specimens of a age higher than the age of patients who typically undergo ligament reconstruction [36] and results are possibly an underestimation of the mechanical properties of younger patients. Effect of age was assessed on 82 patellar tendons taken from donors between 17 and 54 years of age. These tendons were tested at deformation rates of 10% or 100%. The modulus of elasticity was lower only in the older tendon population tested at a deformation rate of 100%. Other biomechanical properties do not appear to be altered with age [3]. Finally, the number of specimens used is small with only 10 specimens allowing preparation of 80 samples.

This study model is based on direct comparison of samples for each specimen which limits potential confounding factors such as soft tissue degeneration, specimens age and conservation process. It provides important information on the biomechanical characteristics of the different layers of the QT for its clinical use as grafts in anterior cruciate ligament reconstruction. But our results are extracted from an Ex-vivo load to failure’s mechanical testing, extrapolation to hamstring quadriceps or patellar tendon behavior in ligament reconstructed patients must be done with caution.

## Conclusion

This study allows us to conclude that all central and median quadriceps tendon deep and superficial layers has interesting biomechanical properties for anterior cruciate ligament reconstructions. No significant difference in material properties was seen between the QT Central and Median layers and ITB or Hamstrings but remain lower in terms of mechanical resistance to PT.

## Data Availability

Not applicable.
